# Correlation between resting state fMRI total neuronal activity and PET metabolism in healthy controls and patients with disorders of consciousness

**DOI:** 10.1002/brb3.424

**Published:** 2015-12-29

**Authors:** Andrea Soddu, Francisco Gómez, Lizette Heine, Carol Di Perri, Mohamed Ali Bahri, Henning U. Voss, Marie‐Aurélie Bruno, Audrey Vanhaudenhuyse, Christophe Phillips, Athena Demertzi, Camille Chatelle, Jessica Schrouff, Aurore Thibaut, Vanessa Charland‐Verville, Quentin Noirhomme, Eric Salmon, Jean‐Flory Luaba Tshibanda, Nicholas D. Schiff, Steven Laureys

**Affiliations:** ^1^ Department of Physics & Astronomy, Brain and Mind InstituteWestern UniversityLondonOntarioCanada; ^2^ Department of Computer ScienceUniversidad Central de ColombiaBogotáColombia; ^3^ GIGA‐Research & Cyclotron Research CentreUniversity of LiègeLiègeBelgium; ^4^ Department of RadiologyWeill Cornell Medical CollegeNew YorkNew York; ^5^ Department of Algology and Palliative CareUniversity Hospital of LiègeLiègeBelgium; ^6^ Brain and Spine InstituteInstitut du Cerveau et de la Moelle épinière (ICM)Hôpital Pitié‐SalpêtrièreParisFrance; ^7^ Brain Innovation B.V.Maastrichtthe Netherlands; ^8^ Department of NeurologyUniversity Hospital of LiègeLiègeBelgium

**Keywords:** Disorders of consciousness, FDG‐PET, fMRI, ICA, metabolism, resting state

## Abstract

**Introduction:**

The mildly invasive 18F‐fluorodeoxyglucose positron emission tomography (FDG‐PET) is a well‐established imaging technique to measure ‘resting state’ cerebral metabolism. This technique made it possible to assess changes in metabolic activity in clinical applications, such as the study of severe brain injury and disorders of consciousness.

**Objective:**

We assessed the possibility of creating functional MRI activity maps, which could estimate the relative levels of activity in FDG‐PET cerebral metabolic maps. If no metabolic absolute measures can be extracted, our approach may still be of clinical use in centers without access to FDG‐PET. It also overcomes the problem of recognizing individual networks of independent component selection in functional magnetic resonance imaging (fMRI) resting state analysis.

**Methods:**

We extracted resting state fMRI functional connectivity maps using independent component analysis and combined only components of neuronal origin. To assess neuronality of components a classification based on support vector machine (SVM) was used. We compared the generated maps with the FDG‐PET maps in 16 healthy controls, 11 vegetative state/unresponsive wakefulness syndrome patients and four locked‐in patients.

**Results:**

The results show a significant similarity with ρ = 0.75 ± 0.05 for healthy controls and ρ = 0.58 ± 0.09 for vegetative state/unresponsive wakefulness syndrome patients between the FDG‐PET and the fMRI based maps. FDG‐PET, fMRI neuronal maps, and the conjunction analysis show decreases in frontoparietal and medial regions in vegetative patients with respect to controls. Subsequent analysis in locked‐in syndrome patients produced also consistent maps with healthy controls.

**Conclusions:**

The constructed resting state fMRI functional connectivity map points toward the possibility for fMRI resting state to estimate relative levels of activity in a metabolic map.

## Introduction

Consciousness is thought to represent an emergent property of cortical and subcortical neural networks (Tononi and Edelman 1998; Laureys 2005; Dehaene et al. 2006; Seth et al. 2011). Clinical research in severely brain injured patients with disorders of consciousness (DOC) is trying to understand the neural causes and failing mechanisms of consciousness (Laureys et al. 2004; Owen et al. 2009; Laureys and Schiff 2012). One of the most severe DOC, the vegetative state (VS, also called unresponsive wakefulness syndrome (UWS) (Laureys et al. 2010)), manifests itself as the persistence of arousal, wakefulness and rest cycles, respiration, and autonomic control, but without external signs of awareness (Ashwal and Cranford 1995; Laureys et al. 2010). In VS/UWS, a metabolic dysfunction as measured by 18F‐fluorodeoxyglucose positron emission tomography (FDG‐PET) was found mostly in a wide frontoparietal network encompassing polymodal associative cortices (Teasdale and Jennett 1974; Teasdale et al. 1983; Kinney and Samuels 1994; Thibaut et al. 2012).

Although FDG‐PET might be considered the most robust neuroimaging technique to clinically investigate severely brain injured patients with DOCs (Stender et al. 2014a,b), important progress has been made in investigating the blood oxygen level dependent (BOLD) signal by functional magnetic resonance imaging (fMRI) (Laureys and Schiff 2012). A variety of strategies has been used in subjects with DOC such as passive paradigms using sensory stimulations (Owen et al. 2005a,b; Coleman et al. 2007; Di et al. 2008) or active tasks, using for example motor imagery (Owen et al. 2006; Boly et al. 2007; Monti et al. 2010; Bardin et al. 2011). However, probably the most appealing clinical approach, for simplicity and non‐invasiveness of acquisition, consists of studying fMRI resting state activity. It intends to measure the patients’ spontaneous activity while lying inside the scanner without performing any specific task or being exposed to any stimulation. During such resting state conditions, human cortex manifests ultra‐slow modulations of neuronal activity reflected both in firing rate modulations of individually isolated cortical neurons, as well as in modulation of high frequency gamma power of local field potentials (He et al. 2008; Nir et al. 2008). These ultra‐slow fluctuations show a remarkable coherence across widespread functionally similar sites and show remarkable reproducibility among subjects (Damoiseaux et al. 2006). Recent studies on spontaneous brain activity revealed that these robust activation patterns correlate to lower (e.g., motor, auditory, visual) and higher order cognitive function (e.g., internal thoughts, language) (Biswal et al. 1995; Lowe et al. 1998; Cordes et al. 2000; Greicius et al. 2003; Fox et al. 2005; Damoiseaux et al. 2006; Nir et al. 2006; Fox and Raichle 2007; Vincent et al. 2007). These results suggest that the study of higher order associative network functionality using resting state fMRI is useful in the absence of patients’ collaboration (Greicius et al. 2004; Boly et al. 2009; Rombouts et al. 2009).

Data driven approaches such as independent component analysis (ICA) (Hyvarinen et al. 2001) applied to spontaneous activity produce a set of independent networks with a particular spatial distribution and a characteristic frequency power spectrum (McKeown et al. 1998; Beckmann et al. 2005; De Luca et al. 2006; Esposito et al. 2008; Perlbarg and Marrelec 2008). They offer the advantage to better isolate physiological artifacts from the neuronal components, and are now being commonly adopted in this field (De Martino et al. 2007; Perlbarg et al. 2007; Birn et al. 2008; Soddu et al. 2012). One important limitation of ICA is that no rules are provided by the methodology for the selection of the independent components, a mandatory step if a quantitative comparison is desired (Seghier et al. 2010). Different procedures have been developed which are based on running a similarity test with templates of the spatial patterns to be recognized (Greicius et al. 2004; Esposito et al. 2005). These procedures can provide satisfying results for healthy subjects or in pathologies with a marginal effect on network spatial patterns. However, when dealing with patients with DOC much more attention is required as the highly deformed brains can significantly alter the network patterns to the extent that they can disappear (Soddu et al. 2011).

In this work we try to overcome the problem of recognizing the different networks by first separating neuronal from artifactual components. As previously introduced in a recent work (Demertzi et al. 2014), in which it was still intended to recognize ten different networks, a support vector machine (SVM) classifier is used to discriminate between neuronal or artifact related independent components (IC). Neuronal ICs are combined in a single scalar map (see Buckner et al. 2009; Cole et al. 2010b). This is done by taking the square root of the absolute *z* maps. The resulting map is then used as a proxy for the resting fMRI neuronal activity. Once this fMRI scalar map has been constructed for each single subject, the same statistical procedures as for comparing metabolic activity maps can be used at the group level allowing quantitative comparisons between healthy subjects and patients. The introduced method is not intending to capture the absolute metabolic activity that only FDG‐PET can provide but gives an estimate of the relative levels of activity. We expect a high correlation between the calculated fMRI total neuronal activity and the metabolic maps across subjects, as well as consistent results when comparing VS/UWS patients versus healthy controls in the two methodologies. If both expectations are confirmed it will evoke the possibility to use fMRI as a possible estimate of the relative metabolic activity levels from MRI acquisitions only.

## Materials and Methods

### Procedure

FDG‐PET and resting fMRI, eyes closed, were obtained in 11 VS/UWS patients (six women, mean age 50 year, SD = 14 year, 10 with non‐traumatic brain injury), four LIS patients (three women, mean age 35 year, SD = 13 year, two non‐traumatic brain injury), see Tables 1 and 2 for demographic and clinical data, and 16 age‐matched healthy controls (six women, mean age = 45 year, SD = 16 year) to the VS/UWS patients’ group. Patients with strongly deformed brains were excluded. (Due to more conservative exclusion criteria and FDG‐PET availability there was an overlap of only one VS and one LIS patient with Soddu, et al., 2012). Informed consent was obtained from all control subjects and from the legal representative of all patients. The study was approved by the ethics committee of the University and University Hospital of Liège in line with the Declaration of Helsinki. Patients’ diagnosis was based on repeated Coma Recovery‐Scale‐Revised assessment (Giacino et al. 2004) prior and following scanning. FDG‐PET and fMRI scannings were within a period of 4 days, behavioral diagnosis was the same on both days. FDG‐PET data were acquired after intravenous injection of 300 MBq of FDG on a Philips Gemini TF PET‐CT scanner as previously described (Bruno et al. 2012; Thibaut et al. 2012). For both PET and fMRI acquisitions, patients were monitored by two anesthesiologists throughout the procedure and visual monitoring assured minimal visual stimulation (e.g., eyes closed, dark room). In order to reduce the influence of not accurate measured radiotracer activity concentrations due to the relatively low image resolution and the limited tissue sampling, phenomenon known as partial volume effect (PVE) ‐ particularly critical when the relative proportion of brain tissue components is altered ‐ a partial volume effect correction was applied to the PET images (Quarantelli et al. 2004). Resting state BOLD data were acquired in the same population on a 3T MR scanner (Trio Tim, Siemens, Germany) with a gradient echo‐planar sequence using axial slice orientation: 32 slices, TE = 30 msec, flip angle = 78^o^, voxel size = 3.0 × 3.0 × 3.0 mm^3^, TR = 2000 msec. 300 volumes were acquired. fMRI and FDG‐PET data were aligned, coregistered and spatially normalized using SPM8 (RRID:nif‐0000‐00343; www.fil.ion.ucl.ac.uk/spm). FDG‐PET data were subsequently smoothed (full‐width half‐maximum of 16 mm). SUV were calculated as previously described (Laureys et al. 2000). For fMRI an initial smoothing of 8 mm was applied before ICA using 30 ICs. Number of components was chosen empirically based on the experience of the authors in application of ICA to resting state fMRI in DOC (Demertzi et al. 2014). See also (Ylipaavalniemi and Vigario 2008) for an investigation of the reliability of solutions found with ICA algorithms. ICA was performed using GIFT (Calhoun et al. 2001). Finally, fMRI motion parameters were calculated as previously described (Soddu et al. 2012): measuring average displacement and average speed during the full acquisition time window.

**Table 1:** Clinical, electrophysiological and structural imaging data of the VS/UWS patients

Clinical features	VS1	VS2	VS3	VS4	VS5	VS6	VS7	VS8	VS9	VS10	VS11
Gender (age, years)	Male (49)	Female (38)	Female (53)	Female (63)	Male (28)	Female (69)	Male (57)	Male (36)	Male (48)	Female (32)	Female (73)
Cause	Cerebro vascular accident	Anoxia	Hypoglycemia	Cerebro vascular accident	Anoxia	Anoxia	Traumatic	Anoxia	Anoxia	Anoxia	Oclusion basilar artery
Time of fMRI (days after admission)	2884	1.730	24	32	93	61	24	2036	140	486	60
Clinical evaluation at time of fMRI	VS	VS	VS	VS	VS	VS	VS	VS	VS	VS	VS
Coma Recovery scale‐Revised total score	7	5	5	6	5	4	1	7	4	5	5
Auditory function	Auditory startle	Auditory startle	Auditory startle	Auditory startle	Auditory startle	None	None	Auditory startle	Auditory startle	Auditory startle	Auditory startle
Visual function	Blinking to threat	None	None	Blinking to threat	None	None	None	None	None	None	None
Motor function	Abnormal posturing	Abnormal posturing	Flexion withdrawal	Abnormal posturing	Abnormal posturing	Flexion withdrawal	None	Abnormal posturing	None	Flexion withdrawal	Flexion withdrawal
Oromotor/Verbal function	Vocalization/Oral movement	Oral reflexive movement	Oral reflexive movement	Oral reflexive movement	Oral reflexive movement	Oral reflexive movement	None	Vocalization/Oral movement	Oral reflexive movement	Oral reflexive movement	Oral reflexive movement
Communication	None	None	None	None	None	None	None	None	None	None	None
Arousal	Eyes open without stimulation	Eyes open without stimulation	Eyes open with stimulation	Eyes open with stimulation	Eyes open without stimulation	Eyes open with stimulation	Eyes open with stimulation	Eyes open without stimulation	Eyes open without stimulation	Eyes open with stimulation	Eyes open with stimulation
EEG											
Background activity	Delta‐theta irregular	Background symmetric rhythm of 8–9 Hz	Low voltage background symmetric rhythm of 3–4 Hz	Irregular symmetric rhythm of 4–5 Hz	Isoelectric activity with eye‐induced artefacts	Diffuse 3–4 hz activity, mainly in frontal regions	Irregular symmetric rhythm of 5–6 Hz, Moderate encephalopathy with brainstem suffering	Background symmetric rhythm of 7 Hz with intermittent slow activity	Predominance of slow bilateral delta activity of low amplitude with burst of slow waves of higher amplitude	Predominance of slow bilateral delta	Predominance of slow bilateral delta activity of low amplitude with burst of slow waves of higher amplitude
MRI											
	Diffuse cortical atrophy. Quadriventricular hydrocephaly	Diffuse cortico‐subcortical atrophy. Diffuse leucoencephalopathy	Brainstem, thalamic and diffuse cortical lesions. Precuneal and subcortical encephalopathy. Diffuse corpus callosum athrophy	Bilateral cerebellar ischemic lesions pronounced on the left side and extended to the vermis and left cerebellar peduncle	Diffuse leukoencephalopaty with bihemispheric cortical ischemic sequels	Diffuse cortico‐sub‐cortical and thalamic lesions. Bilateral hypocampal athrophy	Bilateral cerebellar ischemic lesions pronounced on the left side and extended to the vermis and left cerebellar peduncle	Diffuse athrophy. Quadriventricular hydrocephaly	Periventricular leukoencephalopathy. Cortico‐spinal tract degeneration	Diffuse cortical‐subcortical atrophy. Cortical‐thalamus and basal ganglia ischemic sequels. Periventricular leukoencephalopahty	Ischemic lesions with hemorrhagic infarction in cerbellar hemispheres, right thalamus and right occipital lobe

VS, vegetative state; UWS, unresponsive wakefulness syndrome, fMRI, functional magnetic resonance imaging, EEG, electroencephalography .

**Table 2:** Clinical features of LIS patients

Clinical features	LIS1	LIS2	LIS3	LIS4
Diagnosis	Classical LIS	Functional LIS	Classical LIS	Classical LIS
Gender (age, years)	Male (20)	Female (36)	Female (28)	Female (54)
Etiology	Traumatic	Cerebro vascular accident	Cerebro vascular accident	Traumatic
Time of fMRI (years/months)	4j 5 m	6j 2 m	5j 9 m	5j 10 m

LIS, locked‐in syndrome; fMRI, functional magnetic resonance imaging.

### fMRI components selection

For the neuronal component selection we applied the same procedure as in (Demertzi et al. 2014). A group of 19 independently studied healthy controls (10 women, mean age = 23 year SD = 3 year) was initially used for a supervised learning machine classification between “neuronal” versus “non‐neuronal”. ICs samples were obtained from the ICA decomposition (30 components) of this control group. Data were acquired on a Siemens Magneton 3T MR scanner with a gradient echo‐planar sequence using axial slice orientation: 32 slices, TE = 40 msec, flip angle = 90°, voxel‐size = 3.4 × 3.4 × 3.0 mm^3^, TR = 2460 msec. We combined expert knowledge and non‐linear statistic (machine learning) to build an automatic classification model. The model was based on the IC‐fingerprint, a low level representation of the spatio‐temporal information of each independent component. This feature vector aims to characterize the high energy contributions of low frequency ranges (0.01–0.1 Hz) of the time course and the high sparsity of the spatial map, both properties typically attributed to neuronal sources in resting state (Cordes et al. 2000; Raichle et al. 2001; Daubechies et al. 2009). Additionally, the vector included measures of information content and coherency, important variables to characterize non‐neuronal ICs (De Martino et al. 2007). This feature vector is robust to large geometric deformations, because it is computed on the IC‐spatial distribution without any strong localization prior. A similar representation has been used previously in a multiclass classification setting to characterize six types of ICs (one neuronal and five physiologically noise related (De Martino et al. 2007)). This approach requires the expert to visually classify components in one of these six classes. However, previous studies showed that during the classification process it is not always clear which specific noise class is the most appropriate (Tohka et al. 2008). For this reason, we simplified the labeling task by requiring the expert to classify components in two main classes: “neuronal” and “non‐neuronal”. With this strategy, the classification problem was reframed from a multiclass to a binary classification. We also introduced the category of labeling of “undefined components” to only keep good quality samples for the training process. This category was defined to account for possible limitations of the expert to perform the labeling task (Hartendorp et al. 2012). In this work, the aim was to label a set of components in one of the two biological categories commonly used to classify these components, namely, “neuronal” or “non‐neuronal”. To accomplish this objective, a classifier was trained to distinguish between these two categories. Because the interest was to have good discrimination between these categories, and following a conservative approach, the training considered just “good” samples (sure “neuronal” or “non‐neuronal” samples). As it is impossible to know a priori the true label of one “undefined” component, then the classifier evaluation performance was focused in their capacity to predict the labels in unobserved “good” samples, with the expectation that future samples even “undefined” would be properly assigned to the correct category. First, the 570 ICs taken from our independently studied group of healthy controls (19 × 30) were labeled by an expert using one of the three possible categories: “neuronal” (*n* = 224), “non‐neuronal” (*n* = 248) and “undefined” (*n* = 98). Only samples labeled as “neuronal” and “non‐neuronal” were used during subsequent SVM classification. Two different SVM kernels were used and compared: linear (LIN) and radial basis function (RBF). A tuning of the SVM parameters was performed. The regularization parameter we considered was log_10_C_∈_{‐1,0,…,8} (Hsu et al. 2003). The γ parameter of the SVM‐RBF was varied from 0.1 to 1 with increment steps of 0.1. For the evaluation of the classifier performance a nested leave‐one‐out cross‐validation (LOO‐CV) procedure was used to compute the best set of SVM parameters, and additionally, to provide an unbiased estimate of classifier generalization ability. In this procedure, for each LOO‐CV fold, we excluded all data for a single control subject for the test set, then we repeatedly repartitioned the remaining 18 participants into a validation set (1 control) and training set (17 controls). We selected the optimal parameters for the SVM on the validation set before applying it to the test set.

### fMRI total neuronal activity maps

**Figure 1:**
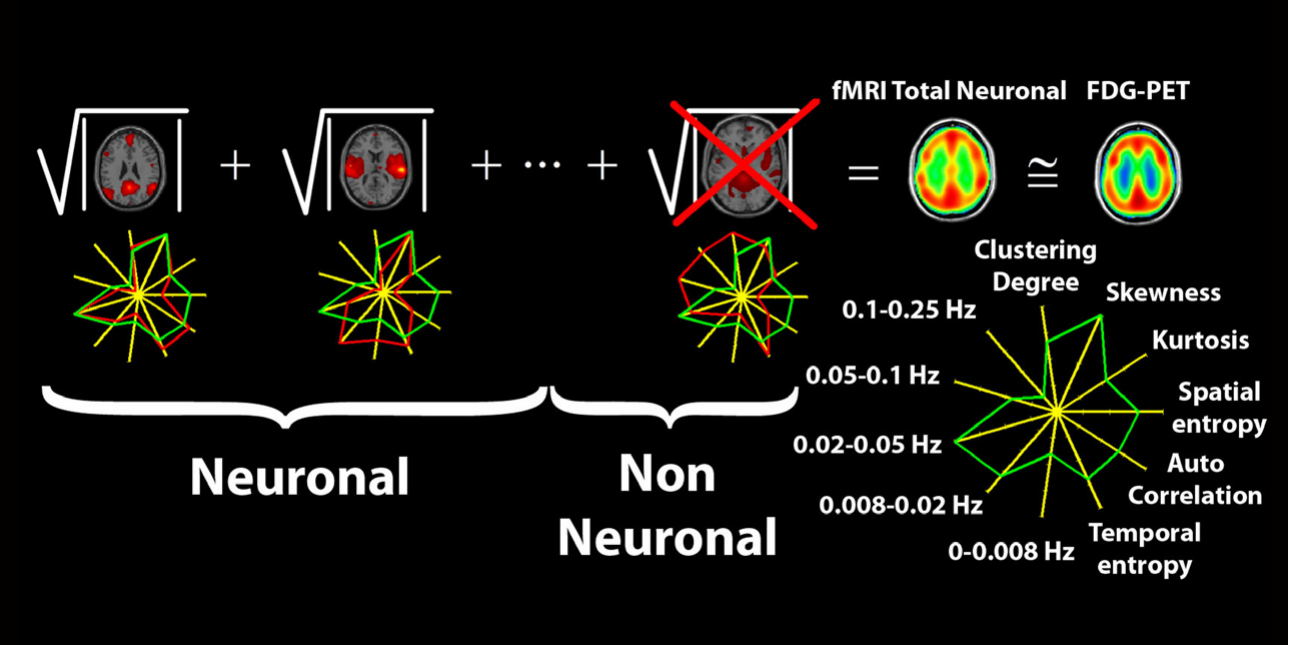
Pictorial description of the methodology used to construct the fMRI total neuronal scalar map starting from automatically selected neuronal independent components. Neuronal independent components were selected based on their fingerprint, which describes for each component, spatial properties of the distribution of the *Z* scores as extracted from the spatial map and temporal properties as extracted from the corresponding time course. The green line on the fingerprint represent the mean values obtained from an independent group of normal volunteers, the red line represents the values observed in the assessed subject.

The area under the Receiver Operating Curve (ROC) was used as performance measurement for the parameter selection (Bradley 1997). The classifier with highest overall classification rate was selected and subsequently used to label neuronal components on the fMRI dataset of the current study. Once the neuronal components were selected, we built a single scalar map for each of the subjects (11 VS/UWS, 4 LIS, and 16 controls) summing voxel by voxel the square root of the absolute value of the *z* maps over the neuronal components (Eq. (1), and described in Fig. 1):

(1) $${\text{fMRI}}_{\mathrm{Tot}}=\sum_{i=1}^{N_{\mathrm{neur}}}\sqrt{|z_{i}|}$$

where *i* is an index for the neuronal components. A subsequent smoothing with a kernel of 16 mm was then applied and a linear correlation value was finally obtained between the calculated fMRI total neuronal map and the corresponding FDG‐PET map. A gray matter mask was created by binarizing the SPM gray matter probability with threshold of 0.1 (Eggert et al. 2012). Correlations were calculated all over the voxels belonging to this mask for both the calculated fMRI total neuronal and FDG‐PET maps.

For the voxel‐based comparison between the VS/UWS and control group we ran a factorial design with two factors, machine and consciousness, each with two levels: respectively FDG‐PET and fMRI total neuronal for the machine factor and healthy controls and VS/UWS patients for the consciousness factor. Both FDG‐PET and fMRI total neuronal scalar maps were proportionally scaled by the global signal before running the group analysis. We considered the following contrasts: (1) FDG‐PET metabolic activity higher in healthy controls with respect to VS/UWS patients; (2) FDG‐PET metabolic activity preserved in VS/UWS patients; (3) fMRI total neuronal activity higher in healthy controls with respect to VS/UWS patients; (4) fMRI total neuronal activity preserved in VS/UWS patients; (5) conjunction of impaired regions in patients common to fMRI and PET and conjunction of preserved regions common to fMRI and PET; (6) higher decrease in FDG‐PET metabolic activity with respect to fMRI total neuronal activity; (7) higher decrease in fMRI total neuronal activity with respect to FDG‐PET metabolic activity. Results were considered significant at *P* < 0.05 false discovery rate corrected for multiple comparisons.

## Results

### fMRI component selection

Two different kernels were used and compared for the selection of neuronal ICs as previously applied in (Demertzi et al. 2014), respectively, a linear (LIN) and a radial basis function (RBF). The SVM‐RBF classifier gave a performance on the training dataset of 0.940 ± 0.003% which was significantly higher than the SVM‐LIN performance of 0.927 ± 0.005% (*P* < 0.01). The SVM‐RBF also provided the best classification accuracy (94%) as compared to visual selection in overall for neuronal ICs. Based on these results, we selected the SVM‐RBF classifier for the task of neuronal ICs selection. fMRI total neuronal activity maps were generated from a total number of 13 ± 4 neuronal ICs in healthy controls, 9 ± 5 ICs in VS/UWS patients and 10 ± 5 in LIS patients. The number of selected neuronal components was not significantly different among the groups even if a trend of fewer ICs was observed in VS/UWS patients (*P* = 0.06 between healthy controls and VS/UWS; *P* = 0.27 for healthy controls versus LIS and *P* = 0.85 for LIS versus VS/UWS).

### fMRI total neuronal activity versus FDG‐PET cerebral metabolism

**Figure 2:**
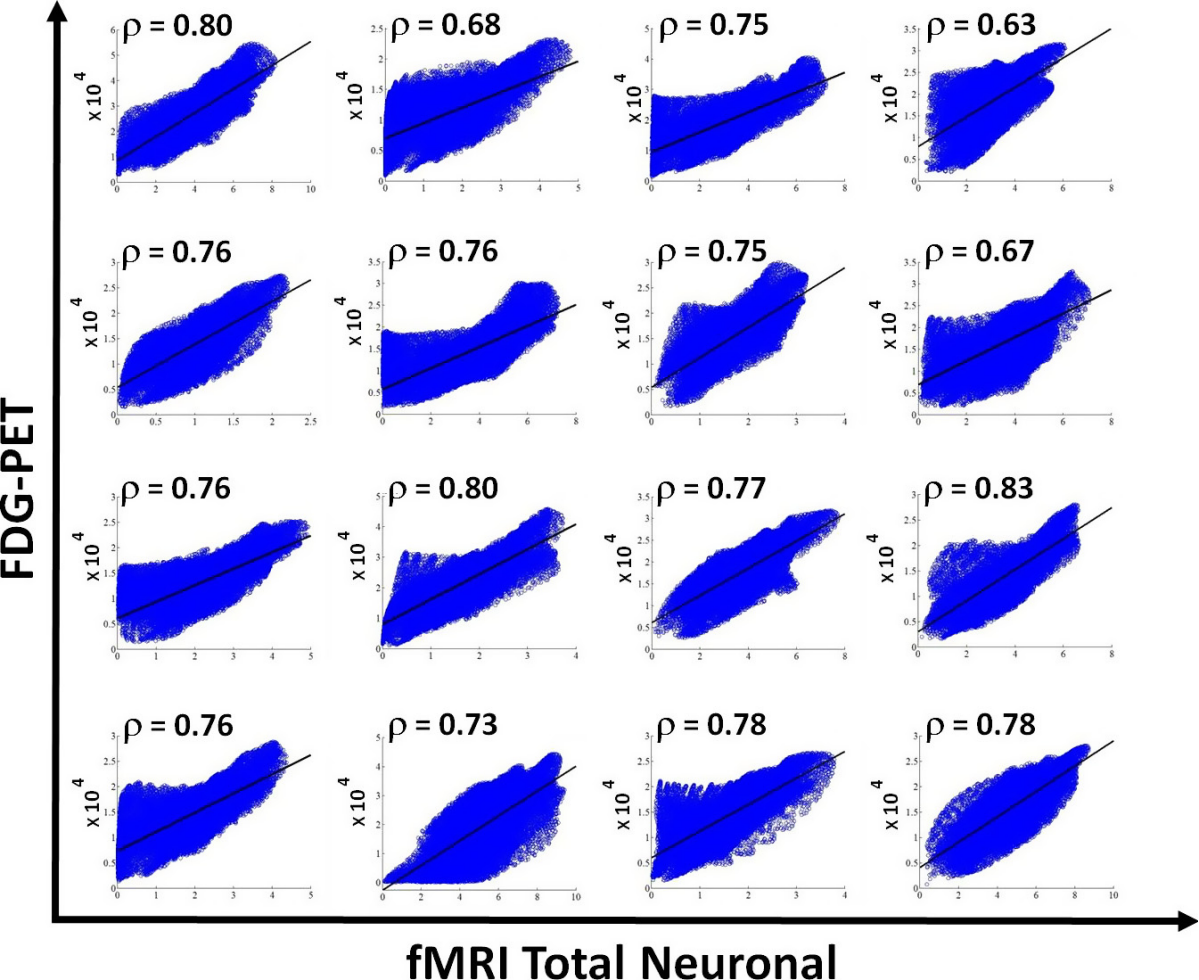
Scatter plots for all the 16 healthy controls showing the correlation between the FDG‐PET after partial volume correction versus the fMRI‐total neuronal activity for voxels belonging to gray matter. Solid line indicates the best linear fit to the data and on the upper left corner of each scatter plot the linear correlation value is reported.

We correlated the fMRI total neuronal activity maps (Eq. (1) in the methods) with the corresponding FDG‐PET metabolic activity maps for all voxels belonging to gray matter. We found for controls a significant (*P* < 0.001) correlation for all voxels of ρ = 0.75 ± 0.05, within the range 0.63–0.83 (see Fig. 2 for the scatter plots reporting FDG‐PET metabolic vs. fMRI total neuronal activity in healthy controls), for VS/UWS patients a significant (*P* < 0.001) correlation of ρ = 0.58 ± 0.09, within the range 0.45–0.76, and for LIS patients a significant (*P* = 0.001) correlation of ρ = 0.67 ± 0.03 ranging from 0.63 to 0.70 (see Figures S1, S2 for the scatter plots reporting FDG‐PET metabolic vs. fMRI total neuronal activity in, respectively, VS/UWS and LIS patients). Correlations between healthy controls and VS/UWS patients were significantly different (*P* < 0.001), the LIS patients group was significantly different from controls (*P* = 0.006), and was not significantly different from VS/UWS (*P* = 0.10). When calculating correlations for all voxels belonging to gray matter using FDG‐PET without performing any partial volume correction we obtained for healthy controls, VS/UWS and LIS, respectively, ρ = 0.74 ± 0.05, ρ = 0.59 ± 0.09, and ρ = 0.67 ± 0.03, which are not significantly different from the correspondent values when performing partial value correction (*P* = 0.71, *P* = 0.80 and *P* = 0.83). More relevant instead was the smoothing we used. Correlations between FDG‐PET partial volume corrected and fMRI total neuronal maps for the smoothing of 8 and 12 mm were, respectively, for healthy controls (ρ = 0.67 ± 0.05, ρ = 0.72 ± 0.05), VS/UWS (ρ = 0.50 ± 0.09, ρ = 0.55 ± 0.09) and LIS (ρ = 0.60 ± 0.03, ρ = 0.65 ± 0.03) with *P* values (*P* < 0.001, *P* = 0.1), (*P* = 0.048, *P* = 0.43) and (*P* = 0.021, *P* = 0.36) when compared to the smoothing of 16 mm.

**Figure 3:**
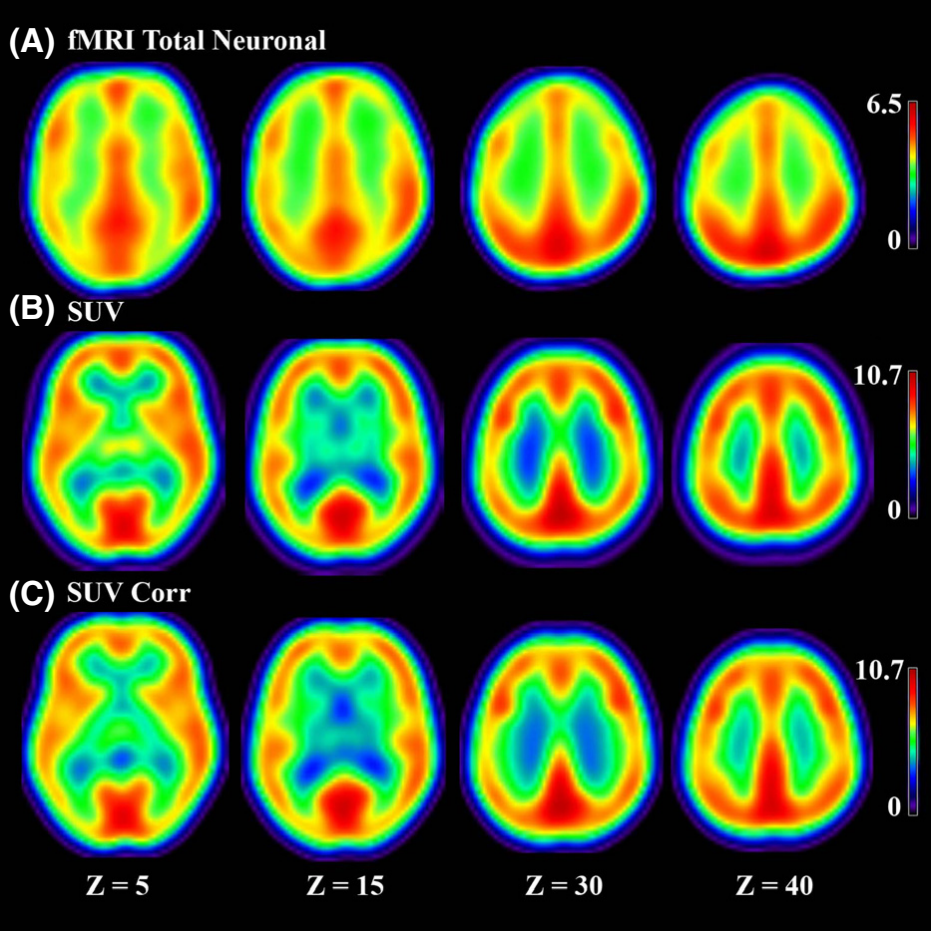
(A) fMRI total neuronal (B) SUV obtained from FDG‐PET without partial volume correction (C) SUV obtained from FDG‐PET after partial volume correction for our group of 16 healthy controls in four coronal slices.

The FDG‐PET standardized uptake value (SUV) metabolic activity, when averaged over gray matter, was higher in healthy controls with a value of 5.5 ± 1.3 as compared to VS/UWS patients with a value of 1.9 ± 1.3 corresponding to a *P* < 0.001 and a global decrease of 35%. SUV value for LIS patients was 5.6 ± 3.2, which was significantly different from VS/UWS (*P* = 0.006). No significant difference was found between LIS and controls (*P* = 0.93). For the fMRI total neuronal activity (see Fig. 3 for a comparison of the average fMRI total neuronal with the average FDG‐PET map in healthy controls, before and after partial volume correction), we obtained, respectively, 3.1 ± 1.3 for healthy controls and 2.2 ± 1.2 for VS/UWS patients, corresponding to a *P* = 0.08, indicating a trend but not a significant reduction in the absolute fMRI total neuronal activity. fMRI total neuronal activity was 2.6 ± 1.4 for LIS patients (*P* = 0.5 compared to controls, and *P* = 0.6 compared to VS/UWS).

**Figure 4:**
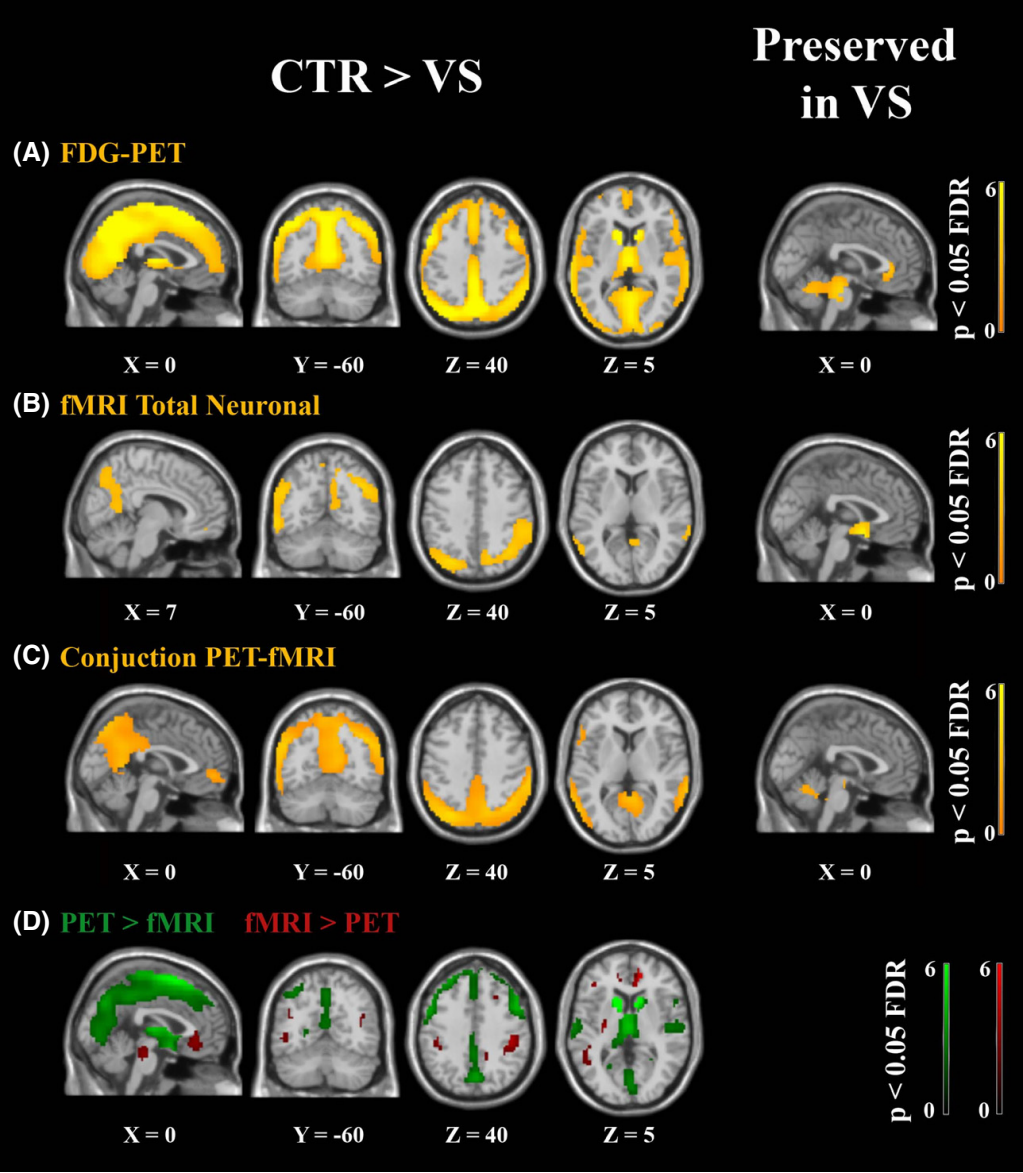
Voxel‐based between‐group analysis for (A) FDG‐PET describing regions (in orange) with higher metabolic activity in healthy controls (CTR) with respect to vegetative state/unresponsive wakefulness syndrome patients (VS/UWS), and regions preserved in vegetative state/unresponsive wakefulness syndrome patients (*P* < 0.05 FDR corrected). (B) fMRI total neuronal activity as for A. (C) conjunction of FDG‐PET and fMRI total neuronal as for a. In green regions with a higher decrease in FDG‐PET with respect to fMRI total neuronal and in red with a higher decrease in fMRI total neuronal with respect to FDG‐PET (for the contrast healthy controls more than vegetative state/unresponsive wakefulness syndrome patients). FDG‐PET was partial volume corrected.

**Figure 5:**
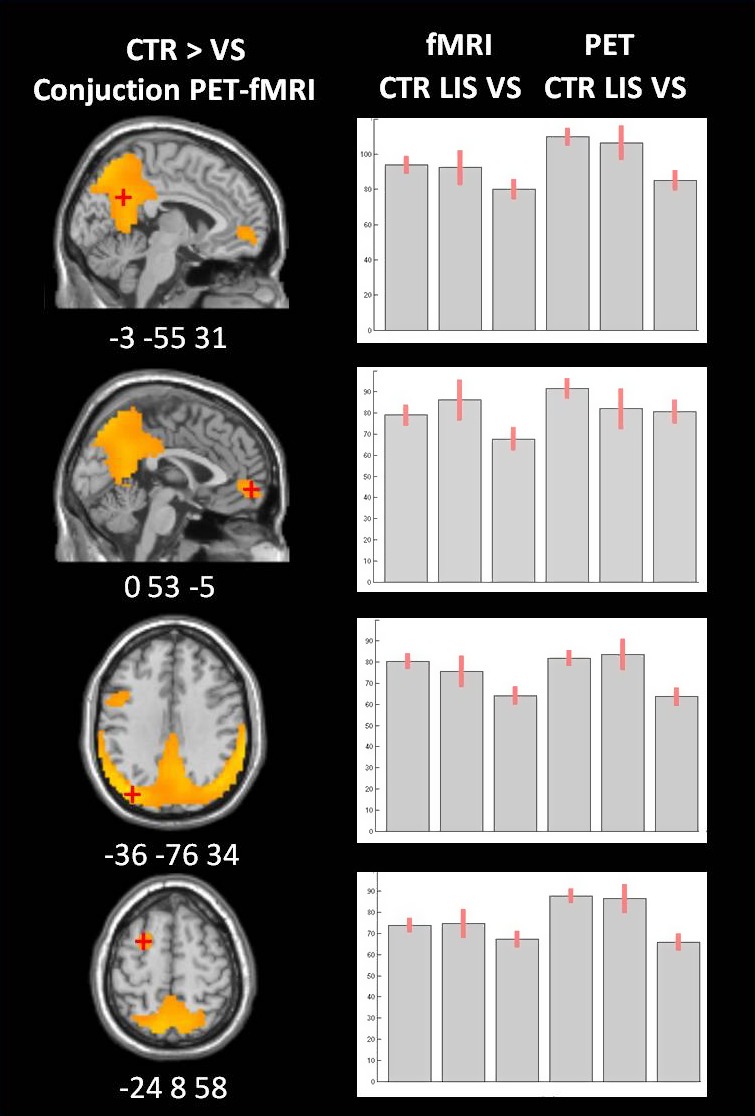
Voxel‐based analysis for the conjunction of FDG‐PET and fMRI total neuronal describing regions with higher metabolic activity in healthy controls compared to vegetative state/unresponsive wakefulness syndrome patients (in orange). The mean *Z*‐scores and 90% confidence interval for the connectivity in the precuneus, the medial frontal gyrus, the left lateral posterior parietal, and the left middle frontal gyrus are visualized for healthy controls, locked in syndrome patients (LIS) and vegetative state/unresponsive wakefulness syndrome patients (VS/UWS) for both fMRI total neuronal and FDG‐PET activity.

**Figure 6:**
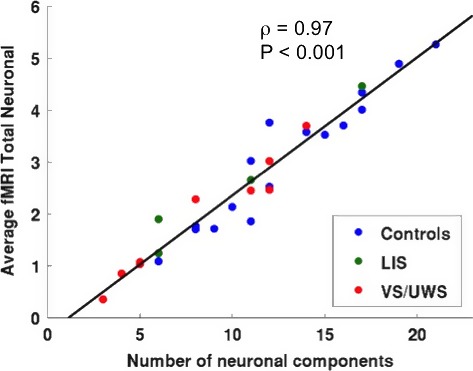
Average fMRI total neuronal activity over gray matter voxels versus total number of neuronal components combining all subjects, healthy controls (CTR), locked‐in (LIS) syndrome patients and vegetative state/unresponsive wakeful syndrome (VS/UWS) patients. Solid line indicates the best linear fit to the data and on the upper right corner the linear correlation value with its corresponding *P*‐value are reported.

When contrasting FDG‐PET metabolic activity proportionally scaled by the global signal in healthy controls versus VS/UWS patients (Fig. 4A), we found that the fronto‐parietal and medial networks (precuneus, medial‐frontal, bilateral posterior parietal, superior temporal, and dorsolateral prefrontal cortices) together with bilateral caudate and thalami appeared as the regions with a significant hypometabolism in VS/UWS patients with respect to healthy controls (*P* < 0.05 FDR corrected; see Table 3). The opposite contrast giving us the regions with preserved FDG‐PET metabolic activity in VS/UWS patients identified the brainstem. When contrasting the fMRI total neuronal activity proportionally scaled by the global signal in healthy controls versus VS/UWS patients (Fig. 4B and Table 3), we also found that regions of the lateral and medial fronto‐parietal networks showed a significant decrease in VS/UWS patients with respect to healthy controls. Intra‐parietal, temporal, and medial orbito‐frontal cortices were the regions showing the most significant decrease. The caudate and thalamus did not show a significant decrease. The hypothalamus appeared as a region with preserved fMRI total neuronal activity in VS/UWS patients. The conjunction analysis of the FDG‐PET metabolic and fMRI total neuronal activities (Fig. 4C and Table 3), when contrasting healthy controls versus VS/UWS patients, confirmed a significant decrease in the fronto‐parietal and medial networks regions (precuneus, medial prefrontal cortex, temporo‐parietal junction, inferior frontal, and medial frontal gyrus). Finally when contrasting the decrease in VS/UWS patients in FDG‐PET with respect to fMRI total neuronal activity (Fig. 4D and Table 3), a significant higher decrease in FDG‐PET with respect to fMRI total neuronal activity was observed in the precuneus, cuneus, insula, caudate, and thalamus. A significant higher decrease was observed in fMRI total neuronal with respect to FDG‐PET metabolic activity in the medial prefrontal cortex and amigdala. The brainstem also appeared in this last contrast being more preserved for VS/UWS patients in FDG‐PET than in fMRI total neuronal. Resting state data of four LIS patients were analyzed even if not included in the group analysis. Figure 5 shows the conjunction analysis of both fMRI and PET for healthy controls, VS/UWS, and LIS patients. Activity of VS/UWS patients was consistently smaller compared to both controls and LIS patients, while LIS patients were comparable to healthy controls. For fMRI, we also calculated two motion parameters (Soddu et al. 2012) measuring average displacement and average speed during the full acquisition time window. We obtained, respectively, a displacement of 0.43 ± 0.25 and a speed of 0.12 ± 0.05 for healthy controls. For VS/UWS patients we found a displacement of 0.60 ± 0.37 (*P* = 0.16 as compared to controls) and a speed of 0.26 ± 0.17 (*P* = 0.006 as compared to controls), while for LIS patients we found a displacement of 0.93 ± 0.46 (*P* = 0.007 as compared to controls, and *P* = 0.17 as compared to VS/UWS) and a speed of 0.10 ± 0.04 (*P* = 0.42 as compared to controls and *P* = 0.09 as compared to VS/UWS). Finally, we plotted the mean value of the fMRI total neuronal versus the total number of neuronal components, Figure 6 considering all the subjects (healthy controls, VS/UVS and LIS patients). The correlation was highly significant with a value ρ = 0.97 and a *P* < 0.001. In Figure S3 we also plotted the correlation between FDG‐PET and fMRI total neuronal versus the total number of neuronal components in each subject. The correlation was not significant with a value ρ = 0.32 and a *P* = 0.089.

**Table 3:** Regions with highest *Z* value appearing in the contrasts healthy controls (CTR) more than vegetative state/unresponsive wakefulness syndrome patients (VS/UWS) and preserved in vegetative state/unresponsive wakefulness syndrome patients for the three cases of FDG‐PET, fMRI total neuronal and conjunction analyses of FDG‐PET and fMRI. Regions with highest *Z* values corresponding to a higher decrease in FDG‐PET with respect to fMRI total neuronal and with a higher decrease in fMRI total neuronal with respect to FDG‐PET (for the contrast healthy controls more than vegetative state/unresponsive wakefulness syndrome patients). Regions are shown in talaraich space

Brain region	*X*,* Y*,* Z*	*T* (df = 51)	*P* (FDR‐corr)
FDG‐PET (CTR > VS/UWS)			
Superior gyrus	−17, 11, 58	3.9	<0.001
Medial frontal cortex	1, 43, 28	3.7	<0.001
Precuneus	0, −64, 36	7.0	<0.001
FDG‐PET (Preserved)			
Brainstem	1, −29, −18	4.7	<0.001
fMRI total neuronal (CTR > VS/UWS)			
Intraparietal cortex	−57, 52, 25	3.7	<0.001
Temporal cortex Left/Right	62, −23, −17	3.3	<0.001
Medial orbitofrontal cortex	0, 45, −10	3.8	<0.001
fMRI total neuronal (Preserved)			
Hipothalamus	−1.8, −3.5, −7.5	3.9	<0.001
PET‐fMRI conjunction (CTR > VS/UWS)			
Precuneus	−0, −56, 13	2.9	<0.001
Intraparietal cortex	46, −40, 46	3.7	<0.001
Inferior frontal gyrus Left/Right	−54, 13, 15	2.0	<0.001
Medial frontal gyrus Left/Right	−48, 12, 26	2.3	<0.001
PET > fMRI (CTR > VS/UWS)			
Precuneus	−0.9, −65, 43	3.2	<0.001
Insula	−43, 19, −3	3.6	<0.001
Caudate	13, 6, 12	5.0	<0.001
Thalamus	−8, −26, 6	4.0	<0.001
PET < fMRI (CTR > VS/UWS)			
Medial prefrotal cortex	6, 40, −7	3.8	<0.001
Brainstem	4, −4, 21	3.6	<0.001
Amigdala	30, −7, 21	4.0	<0.001

FDG‐PET, 18F‐fluorodeoxyglucose positron emission tomography; fMRI, functional magnetic resonance imaging.

## Discussion

There is nowadays a full line of research to understand possible relationship between the FDG‐PET metabolic activity and the fMRI signal (Riedl et al. 2014; Aiello et al. 2015; Nugent et al. 2015). Out of the dynamics of the fMRI BOLD signal it is in fact possible to build in many different ways scalar maps, which can be compared with the metabolic activity maps. All these approaches do not intend to give an absolute estimate of the metabolic activity, like only FDG‐PET can offer, but try to estimate the relative levels of activity between the different regions of the brain, reaching for certain approaches high correlations between the two acquisition techniques when the correlation is limited to gray matter (Aiello et al. 2015). To our knowledge all the approaches presented in the literature use univariate techniques to handle the BOLD signal and build scalar maps. On the other hand, our technique is the very first one using a multivariate approach as ICA to build a single scalar map, taking full advantage of ICA as a recognized tool for the separation of artifacts in the BOLD signal (Griffanti et al. 2014). If our approach, like many others, might be appealing for centers that have no access to FDG‐PET, our main interest in this methodology is that it allows comparison of resting state fMRI in two different populations without the necessity of recognizing the different networks after IC selection (see (Demertzi et al. 2014), for a different approach in which component selection is requested and only subjects in which the component of interest is identified are kept for the group statistical analysis). This new approach permits us to keep all the subjects in the group statistical analysis as long as at least one IC is selected as neuronal and consequently a total fMRI map can be built. Using ICA for analyzing resting state fMRI has been shown to give very relevant insights even in the presence of important artifacts as are commonly found in the BOLD signal recorded in severe brain injured patients with DOC (Demertzi et al. 2014). Mainly for this very relevant aspect we decided to develop an ICA based approach to build our fMRI total neuronal activity maps. However, identification of neuronal components to be included in the fMRI total neuronal activity still remains a challenging task (Cole et al. 2010a).

### fMRI Component selection

A satisfactory solution for the classification of neuronal components at the single individual level can require expert knowledge (De Martino et al. 2007; Tohka et al. 2008), especially for highly deformed brains as commonly observed in patients with DOC. In this work, we took advantage of expert knowledge and non‐linear statistic (machine learning) to build an automatic classification model suitable for this task. Characterization of the neuronal contribution of the resting state signal is one of the most relevant, yet challenging, research question (Griffanti et al. 2014). Removal of the artifactual contribution to the BOLD signal represents a critical step for any resting state based analysis. ICA, which automatically decomposes the resting state signal in sources of neuronal activity and artifactual sources, has been proven to be very successful (Griffanti et al. 2014). However, this approach does not provide any information about the origin of the source, if artifactual or non‐artifactual. Therefore, additional strategies to recognize artifactual components and filter them out should be used. The methodology herein used aims to characterize low frequency components, spatial distribution and coherence as well as the properties of the time series, among other features (Demertzi et al. 2014). This characterization, building a multidimensional fingerprint, was carefully selected to be independent of the spatial pattern of the components. This aspect makes this approach quite suitable in patients with severely affected brains where the spatial pattern can be highly disrupted.

It is important to note that the ground truth used in all these approaches is based on expert knowledge (Griffanti et al. 2014). It is worthy to recall that any classification strategy based or evaluated on these data may reflect expert biases due to the labeling of the specific dataset (Torralba and Efros 2011). Resting state signal in patients with DOC commonly presents components in which characterization (as artifactual or not artifactual) is highly challenging, even for experts. Therefore, a characterization by visual inspection may result in high levels of uncertainty, for these components. In order to reduce the impact of this level of variability in the maps construction, we decided to train the classifier only using components of “clear” origin. Therefore, we could leave the decision about the origin of these uncertain components to the classifier itself. By using this strategy we expect to make a more objective characterization of the components that eventually may contribute to estimate metabolic activity.

### fMRI total neuronal activity versus FDG‐PET cerebral metabolism

Starting from the neuronal classified components we have constructed a single scalar fMRI total neuronal activity map for each subject. These maps were used to compare healthy controls and VS/UWS patients using exactly the same procedure as for the FDG‐PET metabolic activity. First, we observed a high correlation, especially in healthy controls, between the fMRI total neuronal and the FDG‐PET metabolic maps, indicating that it is possible to estimate relative levels of metabolic activity out of resting state fMRI. Even if in fact the relationship between functional connectivity and glucose consumption is still very far from being completely understood, important progress has been done to explain the energetic cost of functional connectivity (Tomasi et al. 2013). This suggests us that the observed high correlation between the fMRI total neuronal and the FDG‐PET metabolic maps is supporting the idea that the BOLD signal is of neuronal origin, with the neuronal contributions isolated in the components, which are selected as neuronal. The ‘total’ in fMRI total neuronal indicates the fact that we use all components of neuronal origin, without focusing on components of some particular spatial pattern. Although the fMRI total neuronal map does not estimate the absolute SUV FDG‐PET values, it can predict the relative weight inside the map. Regions with higher metabolic activity are also the regions for which the time course of the neuronal components can better predict the BOLD signal giving higher spatial map *z* values. Taking the square root of the absolute *z* maps before summing up all neuronal components reduces sparsity (spatial independence), which was initially requested through ICA to decompose the signal (Daubechies et al. 2009) but which is not observed on the FDG‐PET metabolic map. One can in fact expect that the sparsity observed at the neuronal dynamic level, which can distinguish the different networks, is lost the moment one averages over time as an FDG‐PET metabolic activity measure does. It also makes the distribution of the final fMRI total neuronal activity map values more normally distributed as observed for the FDG‐PET metabolic map. Secondly, it is clear that the smoothing we used for both the FDG‐PET and the fMRI total neuronal map is very important for the correlation we reached at the single subject level with the correlation getting significantly higher with the chosen smoothing. On the contrary the use of partial volume correction for FDG‐PET does not seem so important for our analysis. The smoothing, which is commonly suggested when dealing with severe brain injured patients, is in fact probably too high for the partial volume correction having a relevant effect on the analysis. Finally we observed a significant decrease in correlation between the fMRI total neuronal activity map and the FDG‐PET map in VS/UWS and LIS patients. This is most probably due to the difference in motion, as measured mostly by the speed parameter in VS/UWS and in LIS. Motion is producing patches of connected regions especially in the scalp periphery and around the ventricles, which end up corrupting the neuronal contributions.

### Healthy controls versus patients

The group study comparing the two methodologies in VS/UWS patients versus healthy controls is an indication of the possibility to predict diminished or preserved metabolic activity out of fMRI total neuronal activity. Regions appearing in the contrast healthy controls more than VS/UWS patients are interpreted as regions with higher fMRI total neuronal activity and the opposite contrast will give results that can be interpreted as relatively preserved fMRI total neuronal activity. Both FDG‐PET metabolic and fMRI total neuronal maps show significant decreases in the lateral and medial fronto‐parietal networks. The conjunction analysis indeed revealed a significant agreement for precuneus, mesiofrontal, bilateral posterior parietal, superior temporal, and dorsolateral prefrontal cortices, a set of regions which are believed to belong to a network relevant for conscious experience and consciousness (Laureys 2005; Dehaene et al. 2006). Group analysis in this study was done using only the control group and the VS/UWS patient group. The group of LIS patients (*n* = 4) was too small to make any significant conclusions and was therefore not taken into account for the analysis. However, their FDG‐PET as well as fMRI total neuronal activity values are similar to controls while different from VS/UWS patients (see Fig. 5) for the regions in which we observed a decreased in activity for VS/UWS with respect to healthy controls. On the contrary, our technique does not seem to properly estimate metabolic activity in the caudate and thalamus, regions that have not been consistently found in the resting state fMRI literature as part of one or the other neuronal network (Boveroux et al. 2010). This absence of strong connectivity of both the thalamus and the caudate with the cortex, as measured by the BOLD signal in general is a limitation in estimating metabolic activity out of functional connectivity as well. However, the smoothing applied here in order to limit the effects of quite different brain structures (Laureys et al. 1999; Phillips et al. 2011) and to improve the level of similarity between fMRI and PET maps could have mixed different regions in the thalamus or in the caudate washing out possible functional connectivity patterns. Our technique also failed (only a trend was observed) in showing the brainstem as a preserved region when compared to FDG‐PET and as expected for VS/UWS patients in which autonomic control persists (Laureys et al. 2002). It is important to stress here that if only FDG‐PET can offer a quantitative measure of metabolism and our built scalar maps can only capture, with some limitations, the relative levels of metabolic activity, to our knowledge, and especially for FDG‐PET studies in patients suffering severe brain injury with disorders of consciousness, metabolic maps are not always used in their absolute value or the so called standardized uptake value. PET imaging of FDG with arterial blood sampling is in fact ethically tenuous in DOC patients, as the patients cannot give consent to the prolonged invasive procedure (Stender et al. 2014b) and most of the time important approximation are adopted (Stender et al. 2014b) to extract an absolute measure. In many studies, before entering a group comparison, metabolic maps are normalized by either the global signal (Stender et al. 2014a) or the signal of a specific region like it could be the cerebellum or the skull (Fridman et al. 2014), with the first one usually not affected by the pathology and the second one not having any metabolic activity related to brain functioning. Then the moment that normalized maps at the place of the absolute ones are employed, no real advantage in using metabolic maps over scalar fMRI generated maps will be obtained in the analysis, as long as the relative levels of metabolic activity are properly captured. It is also clear that for our analysis becomes essential to have a database of healthy subjects’ fMRI acquisitions as it is commonly done for FDG‐PET when no absolute measures are employed (Stender et al. 2014a).

Finally the high correlation between the average fMRI total neuronal activity and the number of neuronal components is indicating that a reduction in detection of neuronal components, either because of the pathology or the artifacted BOLD signal, will correspond to a lower average fMRI total neuronal activity. At the same time the absence of a significant linear correlation between the correlation of FDG‐PET and fMRI total neuronal with the number of neuronal components, is indicating that some neuronal components more than others are relevant to build up the FDG‐PET map.

In conclusion, we hope that this fully automated procedure of estimating fMRI total neuronal activity from functional connectivity will find applications in (clinical) neuroscience and neuropsychiatry that seek to assess resting state connectivity at the whole brain level, i.e., not restricted to particular sensorimotor or cognitive networks.

## Conflict of Interest

None declared.

## Supporting information


**Figure S1**. Scatter plots for all the 11 VS/UWS patients showing the correlation between the FDG‐PET after partial volume correction versus the fMRI‐total neuronal activity for voxels belonging to gray matter. Solid line indicates the best linear fit to the data and on the upper left corner of each scatter plot the linear correlation value is reported.

**Figure S2**. Same as for Figure S1 for the four LIS patients.

**Figure S3**. Correlation between FDG‐PET, after partial volume correction, and fMRI total neuronal versus total number of neuronal components combining all subjects, healthy controls (CTR), locked‐in (LIS) syndrome patients and vegetative state/unresponsive wakeful syndrome (VS/UWS) patients. Solid line indicates the best linear fit to the data and on the upper right corner the linear correlation value with its corresponding *P*‐value are reported.
