# Supplementary figures and images for: Correlation between resting state fMRI total neuronal activity and PET metabolism in healthy controls and patients with disorders of consciousness
Source: Brain Behav. 2015 Dec 29;6(1):e00424. doi: 10.1002/brb3.424 (PMC4834945; doi:10.1002/brb3.424)

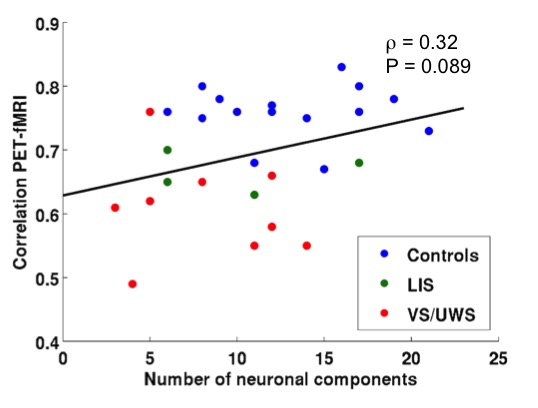

Supplement: Supplementary file 1 — Figure S1. Scatter plots for all the 11 VS/UWS patients showing the correlation between the FDG‐PET after partial volume correction versus the fMRI‐total neuronal activity for voxels belonging to gray matter. Solid line indicates the best linear fit to the data and on the upper left corner of each scatter plot the linear correlation value is reported. [file BRB3-6-e00424-s001.tiff]

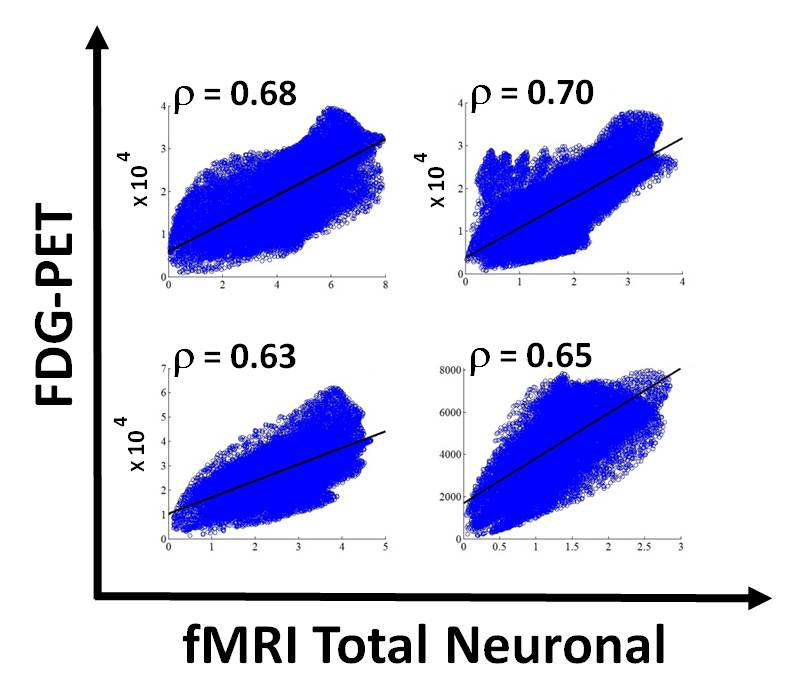

Supplement: Supplementary file 2 — Figure S2. Same as for Figure S1 for the four LIS patients. [file BRB3-6-e00424-s002.tiff]

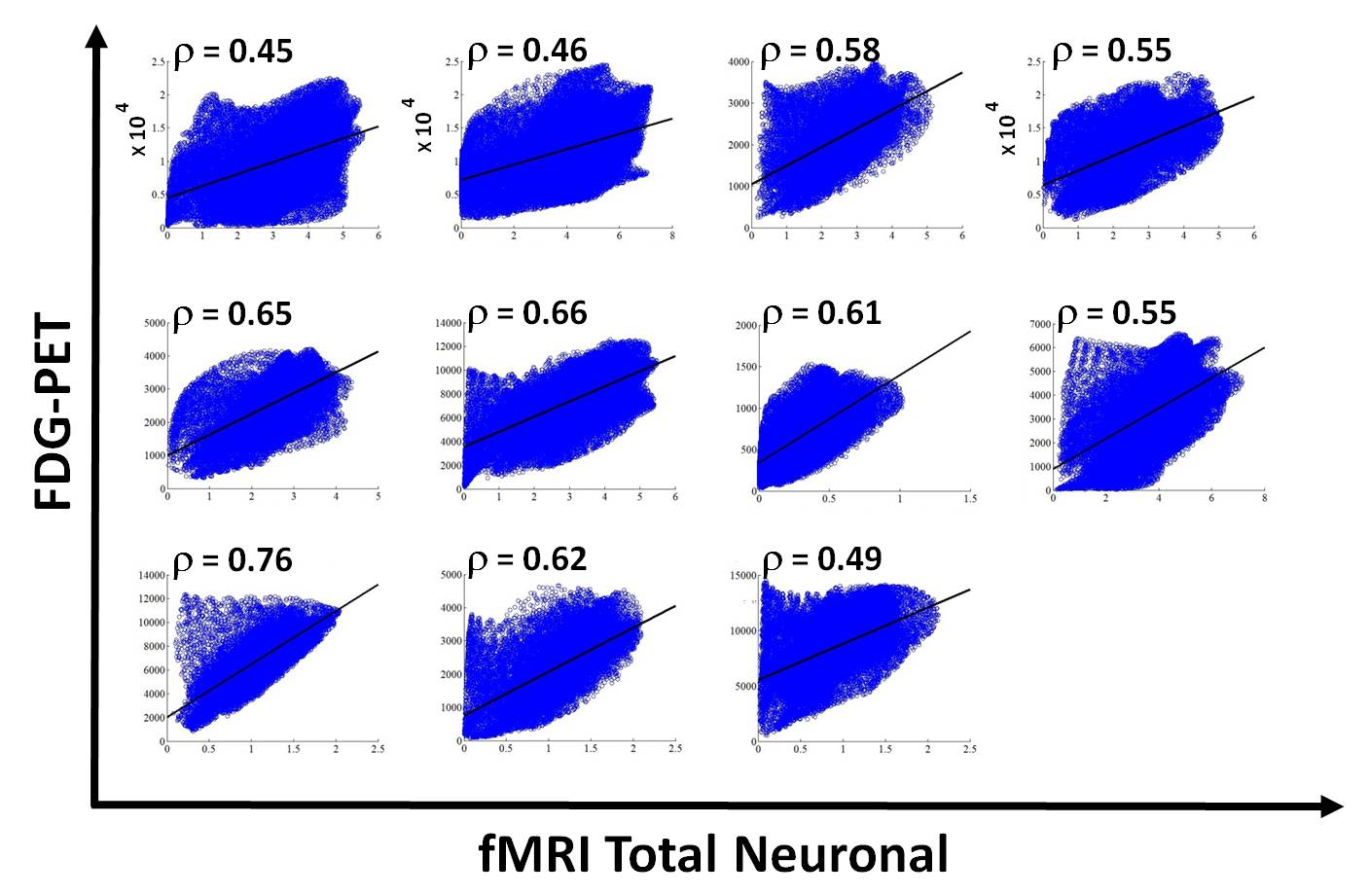

Supplement: Supplementary file 3 — Figure S3. Correlation between FDG‐PET, after partial volume correction, and fMRI total neuronal versus total number of neuronal components combining all subjects, healthy controls (CTR), locked‐in (LIS) syndrome patients and vegetative state/unresponsive wakeful syndrome (VS/UWS) patients. Solid line indicates the best linear fit to the data and on the upper right corner the linear correlation value with its corresponding P‐value are reported. [file BRB3-6-e00424-s003.tiff]
